# How many missed abortions are caused by embryonic chromosomal abnormalities and what are their risk factors?

**DOI:** 10.3389/fgene.2022.1058261

**Published:** 2023-01-04

**Authors:** Xin Li, Han Kang, Huifeng Yin, Tianjiao Liu, Qiannan Hou, Xiaolan Yu, Yuanlin Guo, Wei Shen, Huisheng Ge, Xiaoyan Zeng, Kangmu Lu, Ying Xiong

**Affiliations:** ^1^ Chengdu Women’s and Children’s Central Hospital, School of Medicine, University of Electronic Science and Technology of China, Chengdu, China; ^2^ Chengdu University of Traditional Chinese Medicine, Chengdu, China; ^3^ West China Second University Hospital, West China Women’s and Children’s Hospital, Chengdu, China; ^4^ Chengdu Jinniu Maternal and Child Health Care Hospital, Chengdu, China; ^5^ The Eighth Affiliated Hospital of Sun Yat Sen University, Shenzhen, China

**Keywords:** missed abortion, chromosome abnormality, assisted reproductive technology, advanced age, gene

## Abstract

**Introduction:** Though embryonic chromosome abnormalities have been reported to be the most common cause of missed abortions, previous studies have mainly focused on embryonic chromosome abnormalities of missed abortions, with very few studies reporting that of non-missed abortion. Without chromosome studies of normal abortion samples, it is impossible to determine the risk factors of embryo chromosome abnormalities and missed abortion. This study aimed to investigate the maternal and embryonic chromosome characteristics of missed and non-missed abortion, to clarify the questions that how many missed abortions are caused by embryonic chromosomal abnormalities and what are their risk factors.

**Material and methods:** This study was conducted on 131 women with missed or non-missed abortion from the Longitudinal Missed Abortion Study (LoMAS). Logistic regression analysis was used to identify the association between maternal covariates and embryonic chromosomal abnormalities and missed abortions. Data on the characteristics of women with abortions were collected.

**Results:** The embryonic chromosome abnormality rate was only 3.9% in non-missed abortion embryos, while it was 64.8% in missed-abortion embryos. Assisted reproductive technology and prior missed abortions increased the risk of embryonic chromosome abnormalities by 1.637 (95% CI: 1.573, 4.346. *p* = 0.010) and 3.111 (95% CI: 1.809, 7.439. (*p* < 0.001) times, respectively. In addition, as the age increased by 1 year, the risk of embryonic chromosome abnormality increased by 14.4% (OR: 1.144, 95% CI: 1.030, 1.272. *p* = 0.012). Moreover, advanced age may lead to different distributions of chromosomal abnormality types.

**Conclusion:** Nearly two-thirds of missed abortions are caused by embryonic chromosomal abnormalities. Moreover, advanced age, assisted reproductive technology, and prior missed abortions increase the risk of embryonic chromosomal abnormalities.

## Introduction

Missed abortion, also known as overdue abortion, refers to the fact that the embryo or fetus has died and remains in the uterine cavity without natural discharge, before 12 weeks of gestation (Allen et al., 2022; Herkiloglu et al., 2022). As a special type of spontaneous abortion, missed abortions account for 10%–20% of spontaneous abortions, while 25% of women undergo spontaneous abortion (Practice Committee of the American Society for Reproductive, 2012; Shahine and Lathi, 2015; Mohammad-Akbari et al., 2022). The incidence rate of missed abortions has shown an obvious upward trend in recent years (Alnafisah and Alalfy, 2018; Zhao et al., 2021; Torres-Miranda et al., 2022), which has seriously affected the physical and mental health of the patients, family, and social happiness.

Current research shows that missed abortion is mainly caused by four factors: embryonic factors (chromosome abnormalities), maternal factors (systemic diseases, abnormal reproductive organs, endocrine abnormalities, unhealthy lifestyle, and abnormal immune function), paternal factors (sperm chromosome abnormalities), and environmental factors (Segawa et al., 2017; Zhao et al., 2017; Fang et al., 2018; Fu et al., 2018; Yang et al., 2021a; Gong et al., 2021; He et al., 2021; Liu et al., 2022). Though embryonic chromosome abnormalities have been reported to be the most common cause of missed abortions, previous studies have mainly focused on embryonic chromosome abnormalities of missed abortions, with very few studies reporting that of non-missed abortion. In statistical analysis, the risk factors and their odds ratios of missed abortion can be better determined by comparing the patient characteristics of missed abortion and non-missed abortion. Clarifying the cause of missed abortions is conducive to alleviating the psychological burden of patients, and to carry out reasonable treatment and genetic counseling of these patients for the next pregnancy, by predicting the risk of missed abortions in the subsequent pregnancies (Ashaat and Husseiny, 2012; Hu et al., 2015; Li et al., 2018a).

Chromosome analysis techniques were developed from the earliest karyotype analysis, fluorescence *in situ* hybridization (FISH), chromosome microarray analysis (CMA), and the latest high-throughput sequencing technology (Authors Anonymous, 1988; Dube, 1990; Borgatta et al., 2000; Halder and Fauzdar, 2006; Ashaat and Husseiny, 2012; Segawa et al., 2017; Li et al., 2018b; Dai et al., 2019; Cheng et al., 2021). High-throughput sequencing technology, with its outstanding advantages of high accuracy, throughput, and sensitivity, has been widely used in the field of medical diagnosis (Quintero-Ronderos and Laissue, 2020). In the detection of chromosomal abnormalities in missed abortion villi, high-throughput sequencing technology can detect aneuploidy, large fragment structural abnormalities, chromosome microduplication and microdeletion, and submicroscopic aberrations up to 100 kb, which is relatively superior to other technologies (Ye et al., 2019).

Therefore, in this study, we aimed to investigate the maternal and embryonic chromosome characteristics in missed and non-missed abortion, using high-throughput sequencing technology. Given the findings reported for our cohort, we also aimed to study the impact of maternal characteristics on embryonic chromosomal abnormalities and missed abortions. This data supports the viewpoint that the elimination of altered karyotypes *via* missed abortion represents a strategy to ensure the integrity of karyotype coding (Ye et al., 2019).

## Materials and methods

### Study design and participants

The present study was embedded in the Longitudinal Missed Abortion Study (LoMAS), an ongoing pregnancy and birth cohort study conducted in Chengdu, aiming to determine the relative contributions of genes and the environment to missed abortions (Chinese Clinical Trial Registry: ChiCTR2200060959) approved by the Ethics Committee of the Chengdu Women’s and Children’s Central Hospital (No. 201952). This prospective cohort study was conducted at the Chengdu Women’s and Children’s Central Hospital and included all women with missed abortions as confirmed by ultrasound between March 2021 and December 2021. Written informed consent was obtained from all the participants. This subgroup study included pregnant women who were diagnosed with missed abortions by ultrasound and some matched non-missed abortion women. Non-missed abortions are defined as the normal embryos within 14 weeks of pregnancy terminated pregnancy according to the patient’s requirements and conducted D&C abortions (This is legal in Chinese law). Twin pregnancies were eliminated because it was difficult to separate the villi completely; therefore, women with abortions only in singleton pregnancies were included in this study. Due to the exorbitant rate of abnormal embryos in Perimenopausal women (>45 years old), only women aged 16–45 years were included in the cohort. Women with chromosomal abnormality, chronic metabolic or genetic diseases were not included in the study.

### Data collection

Maternal sociodemographic data (age, height, weight, education, occupation, parity, and mode of conception), lifestyle behaviors before pregnancy (smoking and alcohol use), and preexisting conditions were collected using standardized questionnaires and electronic medical records before D&C abortions. The standardized questionnaire was self-designed for the LoMAS cohort study; detailed information is presented in Supplementary File S1.

### Diagnostic criteria of missed abortion

With the development and popularization of ultrasonic technology, ultrasonic examination has become a common method for clinical diagnosis of missed abortions. According to the French College of Gynaecologists and Obstetricians (CNGOF) (Delabaere et al., 2014), missed abortion can be diagnosed when ultrasound meets any of the following criteria: First, the embryonic head-hip diameter is greater than or equal to 7 mm, and there is no primitive heart tube pulsation; second, the diameter of the gestational sac is more than 25 mm, and no embryo is found; third, ultrasound examination shows that there is no yolk sac in the gestational sac and there is still no embryo with heartbeat after 2 weeks; fourth, ultrasound examination shows that there is a yolk sac in the gestational sac and there is no embryo with heartbeat after at least 11 days.

### Specimen collection and detection process

Villus samples of missed and non-missed abortion patients who terminated pregnancy in the outpatient operating room were collected under strict aseptic conditions. The specimens were transported to the hospital laboratory under refrigeration, where they were washed with normal saline to obtain clean villus tissues. The villi were dried with sterile gauze and frozen at −80° refrigerators.

After all the samples were collected, the villi were processed as follows: villus DNA was extracted using the Universal Genomic DNA Purification Mini Spin Kit (D0063, Beyotime, China). Agarose gel electrophoresis was used to analyze the degree of DNA degradation and RNA contamination, and Qubit was used to detect the total amount and concentration of DNA (standard: total amount of DNA ≥800 ng, DNA concentration ≥10 ng/μL).

Multiplex fluorescent PCR using short tandem repeat (STR) markers (Guangzhou Darui Biotechnology, GuangZhou, China) was performed to exclude maternal cell contamination. High-throughput sequencing for copy number variations (CNV) was performed as previously described. After library preparation, the samples were sequenced using the pair end 150 bp method (PE150) on the Illumina HiSeq platform (Illumina, San Diego, United States) according to the manufacturer’s instructions. Raw image files were processed using BclToFastq (Illumina) for the base calling and raw data generation. The reads were then mapped to the GRCh37/hg19 human reference genome using BWA software. Candidate CNVs were classified using a five-tiered system according to a joint consensus recommendation of the American College of Medical Genetics and Genomics (ACMG) and the Clinical Genome Resource (ClinGen).

### Statistical methods

All statistical analyses were performed using SPSS version 25.0 (IBM Corp., Armonk, NY, United States). The chi-squared or Fisher’s exact test was used to assess categorical data, which were reported as counts and percentages. The means and standard deviations of continuous variables were calculated using the Student’s *t*-test, one-way analysis of variance, or the non-parametric test. Binary logistic regression analysis was used to detect the influence of women’s characteristics on missed abortions and embryonic chromosome abnormalities. Covariates were selected according to the different variables in univariate analysis and the factors reported in previous studies that would affect missed abortion or embryonic chromosome abnormality. All tests were two-tailed, and *statistical significance was set at p < 0.05*.

## Results

**FIGURE 1:**
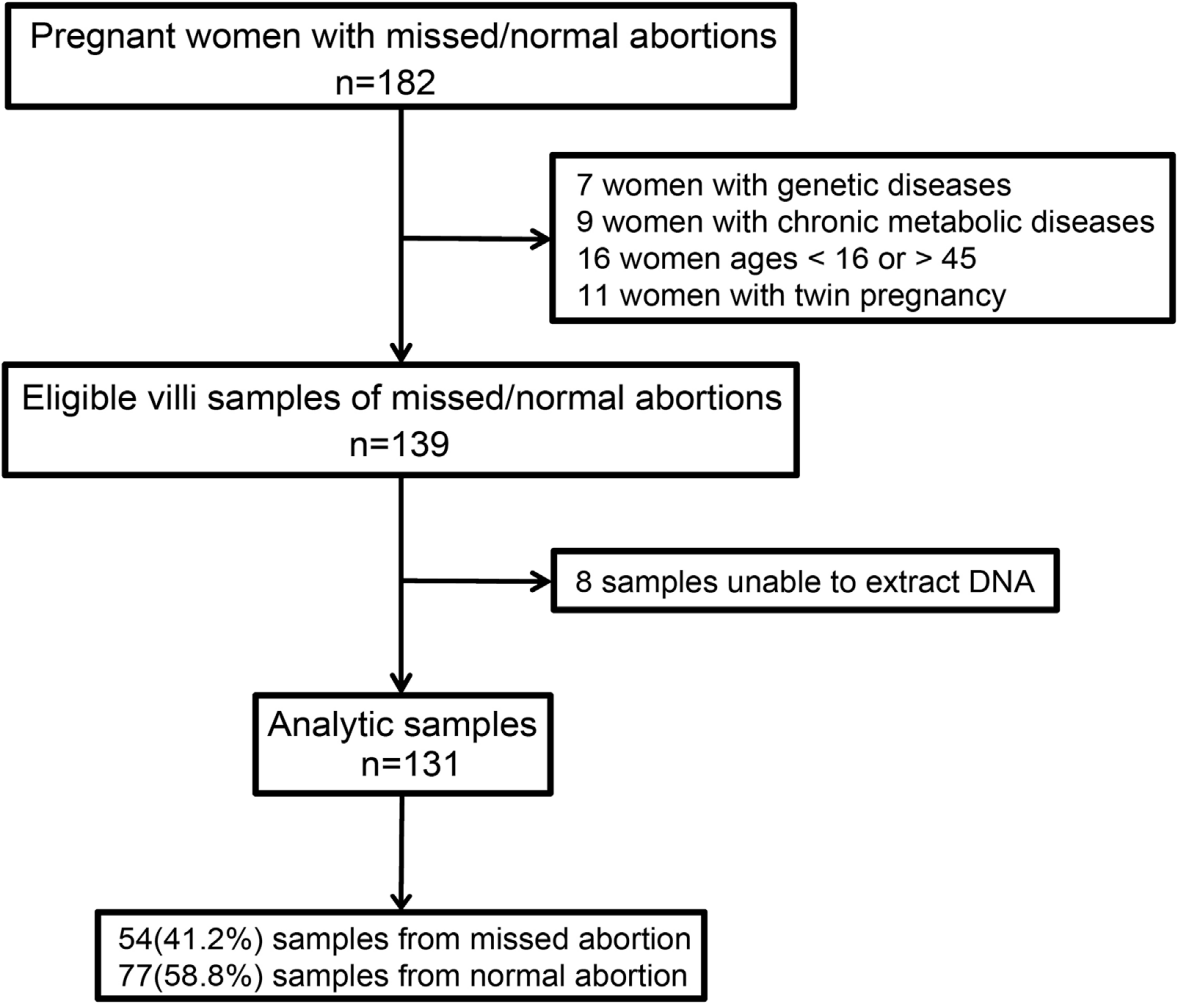
The selection process for this study.

The selection process of the study population is shown in Figure 1. A total of 171 women with missed or non-missed abortion were initially recruited into this subgroup study as part of the LoMAS study. After excluding patients who did not match the inclusion criteria and cases of failure to extract embryonic DNA, the final analysis included 131 women with missed or non-missed abortion. Descriptive data of the study participants are shown in Table 1. The average patient age at delivery was 28.34 ± 5.48 years, and the average gestational age was 8.93 ± 1.85 weeks. Furthermore, 19.1% of patients conceived *via* assisted reproductive technology (ART), and 96.2% of patient parity was less than or equal to two due to the Chinese previous two-child policy. Finally, 54 (41.2%) missed abortions and 77 (58.8%) normal abortion cases were included in the analysis.

**TABLE 1:** Description of the abortion women’s characteristics.

Variables	Total
Number	131
Age (year)	28.34 ± 5.48
Pre-pregnancy BMI (kg/m2)	22.47 ± 3.29
Mode of conception	
Natural conception	106(80.9%)
Assisted reproductive technology	25(19.1%)
Type of abortions	
Missed abortion	54(41.2%)
Non-missed abortion	77(58.8%)
Conception season	
Summer/autumn	59(45.0%)
Winter/spring	72(55.0%)
Gestational age (week)	8.93 ± 1.85
Gravidity	
1	32(24.4%)
2	42(32.1%)
3	27(20.6%)
4+	30(22.9%)
Parity	
0	64(48.9%)
1	42(32.1%)
2	20(15.2%)
3+	5(3.8%)

**FIGURE 2:**
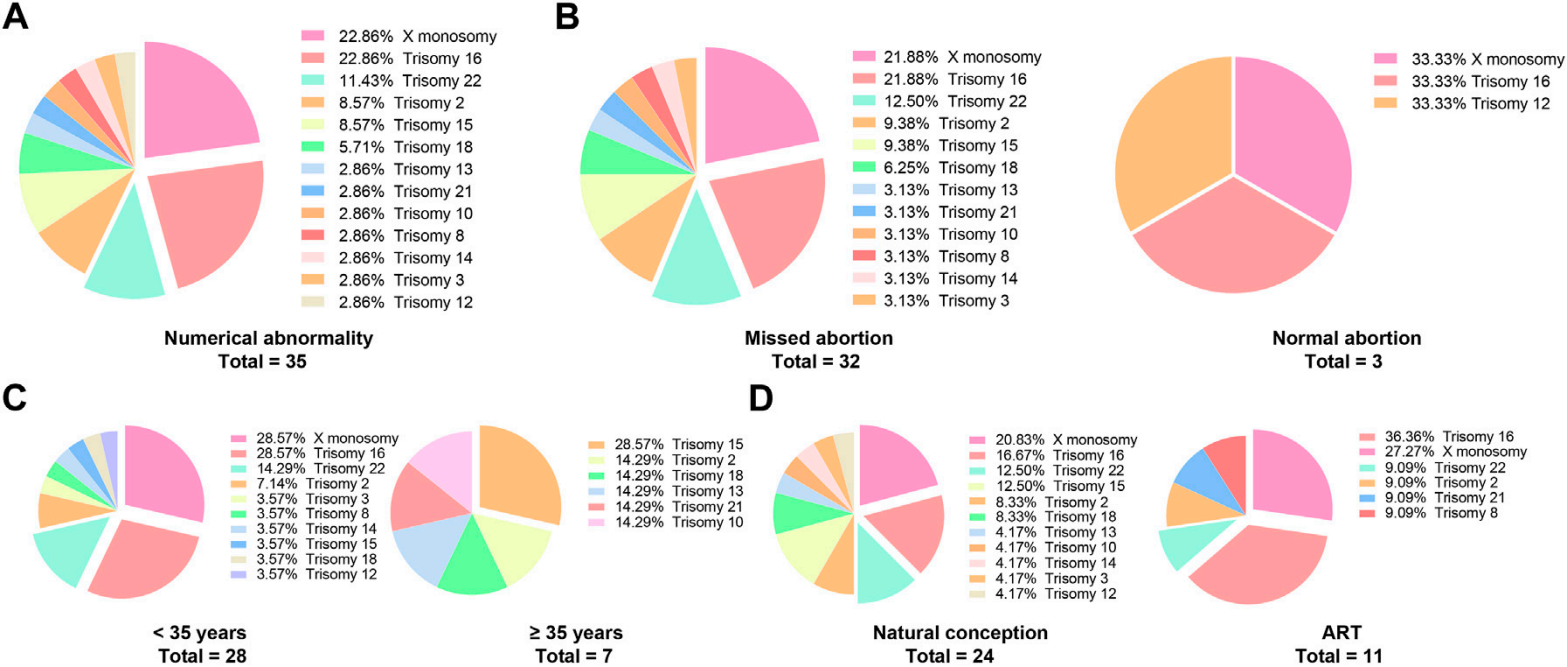
The types of specific chromosomal abnormalities. **(A)** It shows the types of all the chromosomal numerical abnormalities of the aborted embryos; **(B–D)** According to missed abortion, maternal age and ART, the types of chromosome abnormalities were displayed.

In the patients with missed abortions, nearly two-third of the patients whose embryos were accompanied by chromosome abnormality, 25.9% had conceived *via* ART, and 13.0% had a previous missed abortion; this group with higher age and pre-pregnancy BMI had a higher incidence of antenatal bleeding but less parity. Specific abnormal chromosomal types are shown in Figure 2. The top five prevalent chromosomal abnormalities were as follows: 22.86% X monosomy, 22.86% trisomy 16, 11.43% trisomy 22, 8.57% trisomy 2, 8.57% trisomy 15. These five types of chromosomal abnormalities account for three-quarters of all chromosomal abnormalities (Tables 2,3).

**TABLE 2:** Description of the abortion women’s characteristics by type of abortions.

Variables	Missed abortion	Non-missed abortion	*p*-value
Number	*N* = 54	*N* = 77	
Age (year)	29.74 ± 5.03	27.35 ± 5.60	0.013^a^
Pre-pregnancy BMI (kg/m2)	23.30 ± 3.51	21.92 ± 3.38	0.025^a^
Mode of conception			0.004^b^
Natural conception	40 (74.1%)	66 (85.7%)	
ART	14 (25.9%)	11 (14.3%)	
Gestational age (week)	10.23 ± 1.69	8.02 ± 1.36	<0.001^a^
Smoking	4 (7.4%)	2 (2.6%)	0.184^b^
Conception season			0.829^b^
Summer/autumn	23 (42.6%)	36 (46.8%)	
Winter/spring	31 (57.4%)	41 (53.2%)	
Gravidity	3 (2)	3 (2)	0.644^c^
Parity	0 (2)	1 (2)	0.006^c^
Number of prior D&C abortions	2 (2)	2 (2)	0.946^c^
Prior missed abortion	7 (13.0%)	1 (1.3%)	0.019^d^
Falling ill during pregnancy	4 (7.4%)	0 (0%)	0.275^d^
Taking special drugs during pregnancy	2 (3.7%)	1 (1.3%)	0.312^d^
Exposure to hazardous substances	1 (1.8%)	0 (0%)	0.986^d^
TORCH infection	0 (0%)	0 (0%)	1.000^d^
Antenatal bleeding	8 (14.8%)	6 (7.8%)	0.203^b^
Chromosome abnormality	35 (64.8%)	3 (3.9%)	<0.001^d^

BMI, body mass index; ART, assisted reproductive technology, D & C dilation and curettage.

^a^ Average and standard deviation. Student’s *t*-Test.

^b^ Number (percentage). Chi-squared Test.

^c^ Median (interquartile range). Kruskal-Wallis Test.

^d^ Number (percentage). Fisher Exact Test.

**TABLE 3:** Description of the chromosome of villi by type of abortions.

Variables	Missed abortion	Non-missed abortion	*p*-value
Number	*N* = 54	*N* = 77	
Chromosome abnormality	35(64.8%)	3(3.9%)	<0.001^a^
Numerical abnormality	32(59.3%)	3(3.9%)	<0.001^a^
Structural abnormality	3(5.5%)	0(0%)	0.766^a^
Microdeletion			
>0.2 Mb	15(27.8%)	25(32.5%)	0.874^b^
>2 Mb	7(13.0%)	12(15.6%)	0.828^b^
Microdupliction	5(9.3%)	4(5.2%)	0.301^a^

^a^ Number (percentage). Fisher Exact Test.

^b^ Number (percentage). Chi-squared Test.

A significant difference in the patient’s age, pre-pregnancy BMI, mode of conception parity, prior missed abortion, and embryonic chromosome abnormality was found between the missed and normal abortion groups (Tables 2,3). Binary logistic regression showed that missed abortions were significantly associated with age, mode of conception, and parity (OR: 0.691, 95% CI: 0.500, 0.955. *p* = 0.025), prior missed abortions, and embryonic chromosomal abnormalities, but were not found to be correlated with BMI, smoking, conception season, gravidity, D&C abortions. Notably, due to too few samples in falling ill, taking special drugs, and exposure to hazardous substances during pregnancy, we cannot clearly infer their relationship with missed abortion. ART, prior missed abortions, and embryonic chromosome abnormalities increased the risk of missed abortions by 2.110 (95% CI: 1.395, 5.598. *p* = 0.034), 3.040 (95% CI: 1.068, 8.654. *p* < 0.001) and 16.352 (95% CI: 11.230, 40.409. *p* < 0.001) times, respectively. Moreover, as the age increased by 1 year, the risk of missed abortion increased by 15% (OR: 1.150, 95% CI: 1.055, 1.254. *p* < 0.001) (Table 4).

**TABLE 4:** Association between the maternal covariates and missed abortion.

Variables	EXP(B)	95% CI	p-value
Maternal age (year)	1.150	(1.055,1.254)	<0.001
Pre-pregnancy BMI (kg/m2)	0.856	(0.413,2.775)	0.676
Mode of conception	2.110	(1.395,5.598)	0.034
Smoking	0.378	(0.121,1.181)	0.094
Conception season	1.005	(0.984,1.025)	0.652
Gravidity	0.987	(0.760,2.014)	0.328
Parity	0.691	(0.500,0.955)	0.025
Number of prior D&C abortions	0.618	(0.033,1.495)	0.747
Prior missed abortion	3.040	(1.068,8.654)	<0.001
Falling ill during pregnancy	0.992	(0.742,1.327)	0.958
Taking special drugs during pregnancy	0.975	(0.644,1.477)	0.906
Exposure to hazardous substances	0.979	(0.911,1.052)	0.556
Antenatal bleeding	0.998	(0.991,1.005)	0.612
Embryonic chromosome abnormality	16.352	(11.230,40.409)	<0.001

Moreover, microdeletions and microduplications of embryonic chromosomes were analyzed in our cohort using high-throughput sequencing technology. Although the embryonic chromosome microduplication rate in the missed abortion group (9.3%) was higher than that in the normal abortion group (5.2%), there was no significant difference between the two groups (Table 3). The likely pathogenic chromosomal deletions and duplications in this study are shown in Table 5.

**TABLE 5:** Likely pathogenic and pathogenic CNVs detected by high-throughput sequencing technology.

No.	Group	Localization	Deletion/duplication	Length	Likely pathogenic/pathogenic
1	Non-missed abortion	chr19p13.3(2505001_4925001)	Defection	2.420Mb	Possible
2	Non-missed abortion	chr 19p13.3(3265001_5025001)	Defection	1.760Mb	Possible
3	Non-missed abortion	chr 16q24.2q24.3(87570802_90124384)	Defection	2.554Mb	Possible
4	Non-missed abortion	chr 19p13.3(3205001_4485001)	Defection	1.280Mb	Possible
5	Non-missed abortion	chr 19p13.3(2465001_5025001)	Defection	2.560Mb	Possible
6	Non-missed abortion	chr 19p13.3(3835001_4345001)	Defection	0.510Mb	Possible
7	Non-missed abortion	chr 17q23.2q25.3(61480849_80884050)	Loss/gain	19.403M	Pathogenic
8	Non-missed abortion	chr 1q24.1q32.1(166944646_198754646);	Repeation	31.810M	Possible
		chr 1p31.1p21.1(70193083_105128907)		34.936M	
9	Missed abortion	chr 19p13.3(3855001_4665001)	Defection	0.810Mb	Possible
10	Missed abortion	chr 19p13.3(2965001_4195001)	Defection	1.230Mb	Possible
11	Missed abortion	chr 19p13.3(2465001_4885001)	Defection	2.420Mb	Possible
12	Missed abortion	chr 19p13.3(2975001_4995001)	Defection	2.020Mb	Possible
13	Missed abortion	chr 18p11.32p11.31(125001_6795001)	Defection	6.670Mb	Pathogenic

**FIGURE 3:**
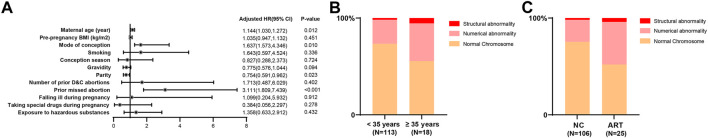
The maternal risk factors for embryonic chromosome abnormalities. **(A)** Binary logistic regression showed that ART and prior missed abortion increased the risk of embryonic chromosome abnormality by 1.6 and 3.1 times, respectively. Besides, with an increase in age by 1 year, the risk of embryonic chromosome abnormality increased by 14.4%; **(B)** 28 (24.8%) women had embryonic chromosomal numerical abnormalities and two (1.8%) had embryonic structural abnormalities in the normal age group, while seven (38.9%) had embryonic chromosomal numerical abnormalities and one (5.6%) had embryonic structural abnormalities in the advanced maternal age group; **(C)** 24 (22.6%) women had embryonic chromosomal numerical abnormalities and two (1.9%) had embryonic structural abnormalities in the natural conception group, whereas 11 (44.0%) had embryonic chromosome numerical abnormalities and one (4.0%) had embryonic structural abnormalities in the ART group.

Considering that nearly two-thirds of the embryos were accompanied by chromosomal abnormalities in women with missed abortions, binary logistic regression was used to analyze the factors influencing embryonic chromosome abnormalities. The results showed that embryonic chromosome abnormalities were significantly associated with age, mode of conception, and parity (OR: 0.754, 95% CI: 0.591, 0.962. *p* = 0.023) and prior missed abortions. ART and prior missed abortions increased the risk of embryonic chromosome abnormalities by 1.637 (95% CI: 1.573, 4.346. *p* = 0.010) and 3.111 (95% CI: 1.809, 7.439. *p* < 0.001) times, respectively. In addition, as age increased by 1 year, the risk of embryonic chromosome abnormality increased by 14.4% (OR: 1.144, 95% CI: 1.030, 1.272. *p* = 0.012) (Figure 3A).

In our cohort, 28 (24.8%) women had embryonic chromosomal numerical abnormalities and two (1.8%) had embryonic structural abnormalities in the normal age group, while seven (38.9%) had embryonic chromosomal numerical abnormalities and one (5.6%) had embryonic structural abnormalities in the advanced maternal age group (Figure 3B). Moreover, 24 (22.6%) women had embryonic chromosomal numerical abnormalities and two (1.9%) had embryonic structural abnormalities in the natural conception group, whereas 11 (44.0%) had embryonic chromosome numerical abnormalities and one (4.0%) had embryonic structural abnormalities in the ART group (Figure 3C).

Considering that the incidence of embryonic chromosomal abnormalities is significantly increased in the advanced maternal age and ART groups, further analysis focused on the effects of age and ART on the types of embryonic chromosome abnormalities. Surprisingly, the distribution and proportion of chromosome abnormalities in the ART group were not significantly different from those in the natural conception group, while there was an evidently different type and distribution of chromosome abnormalities between the normal and advanced maternal age groups (Figures 2B–D).

## Discussion

In this prospective preliminary study, we investigated the maternal and embryonic chromosome characteristics in missed and non-missed abortion using high-throughput sequencing technology. Given the findings reported for our cohort, we found effects of maternal age, ART, prior missed abortion on embryonic chromosome abnormality, and missed abortion.

Previous studies have shown that the incidence of embryonic chromosome abnormalities in missed abortion was 50%–60% (Delabaere et al., 2014; Yang et al., 2021b; Voorhies et al., 2022), which is consistent with the 64.8% reported in our study. In addition, we characterized embryonic chromosomes from non-missed abortion, which were not included in previous studies. This is crucial because the chromosomal abnormality rate of missed abortions is 64.8%, which cannot be attributed to embryonic chromosome abnormalities alone. The “cause and effect” relationship between missed abortion and embryonic chromosome abnormality is undefined when the rate of embryonic chromosome abnormalities in normal abortion has not been detected. In our cohort, the chromosome abnormality rate was only 3.9% in non-missed abortion embryos, while it was 64.8% in missed abortion embryos. Embryonic chromosome abnormality increased the risk of missed abortion by 16.352 times. Based on this result, it can be concluded that embryonic chromosomal abnormalities are the main cause of missed abortions. Recently, the concept of karyotype coding is proposed to illustrate the importance of the normal karyotype, as any altered karyotype can alter the genomic network, some of which is closely associated with disease conditions (Ye et al., 2019). Thus, the high rate of missed abortion can effectively eliminate the altered genome systems (Gorelick and Heng, 2011).

Previous studies have shown that conception with the help of ART may result in a higher rate of missed abortions and embryonic chromosome abnormalities (Askerov, 2016; Li et al., 2018b), which is consistent with our study. After the implementation of Chinese two-child and three-child policies (Chen et al., 2022; Long et al., 2022), many women who cannot get pregnant naturally due to previous tubal ligation or advanced age get pregnant by ART. Further research is necessary to clarify the relationship between ART and embryonic chromosome abnormalities to promote the development of ART technology.

In our cohort, with an increase in maternal age, the rate of missed abortions and embryonic chromosome abnormalities gradually increased (Yang et al., 2021a; Salman et al., 2021; Allen et al., 2022), which is consistent with previous studies. However, although ART and advanced age increase the incidence of chromosomal abnormalities, the types of chromosomal abnormalities are different. The distribution and proportion of chromosomal abnormalities in the ART group were not significantly different from those in the natural conception group, while there was an evidently different type and distribution of chromosomal abnormalities between the normal age and advanced maternal age groups. However, due to the relatively limited sample size in this study, it cannot be concluded that advanced age may lead to different distributions of chromosomal abnormalities. Further research is needed to determine the effect of advanced age on the localization of embryonic chromosomal abnormalities.

Based on our study, previous missed abortions may increase the risk of missed abortions in subsequent pregnancies. To a certain extent, this is consistent with the lower risk of missed abortions in women who have successfully delivered in the past. Embryo chromosome abnormalities arise mainly due to sperm or egg chromosome abnormalities or the influence of external environmental factors in early pregnancy (Del Carmen Nogales et al., 2017; Shen et al., 2019; Wang et al., 2021). Some of these factors are similar in two pregnancies in the same woman, which means that if a woman has two or more missed abortions, both partners need to have a more detailed pre-pregnancy examination before the next pregnancy. Moreover, although some microdeletions and microduplications of embryonic chromosomes have been reported to lead to missed abortion (Cui et al., 2021; Familiari et al., 2021; Kokkonen et al., 2021; Baba et al., 2022; Yue et al., 2022), and the chromosome microduplication rate of the missed abortion group was higher than that of the normal abortion group in our cohort, there was no significant difference between the two groups.

The merits of our study include the inclusion of a specialized study population. Study participants were screened using strict inclusion and exclusion criteria. Non-singleton pregnant women were excluded because it was difficult to separate the villi completely. Additionally, we collected maternal sociodemographic data and carried out chromosome analysis of both missed- and non-missed abortion women, resulting in a comprehensive study design. Because of the difficulty in obtaining normal aborted embryo villi and the high cost of high-throughput sequencing technology, obtaining embryonic villi specimens and detecting them is time-consuming and costly. It is relatively challenging to recruit participants, collect specimens, detect embryonic chromosomes, and follow up for 1 year.

This preliminary study has significantly contributed to our understanding of the impact of maternal characteristics on embryonic chromosome abnormalities and missed abortions, but also has several limitations that need to be acknowledged. First, compared to some epidemiological surveys, the sample size in this study was relatively modest. Second, the 1 year follow-up of their next pregnancy in women with missed abortions is still ongoing; these data will be reported after finishing the follow-up. Third, due to China’s geomorphic and ethnic diversity, missed abortion-related variables, such as living region altitude and nationality, were limited. To further understand the relevant maternal characteristics of embryonic chromosome abnormalities and missed abortions in China, a large-scale study involving more regions and nationalities, conducted in multiple centers, is required.

## Conclusion

In conclusion, this study suggests that nearly two-thirds of missed abortions are indeed caused by embryonic chromosomal abnormalities. Moreover, advanced age, ART, and prior missed abortions increase the risk of embryonic chromosomal abnormalities.

## Data Availability

The original contributions presented in the study are included in Supplementary Data Sheet S2. Further inquiries can be directed to the corresponding authors.
