# Supplementary material for: How many missed abortions are caused by embryonic chromosomal abnormalities and what are their risk factors?
Source: Front Genet. 2023 Jan 4;13:1058261. doi: 10.3389/fgene.2022.1058261 (PMC9846508; doi:10.3389/fgene.2022.1058261)
Supplement: Supplementary file 1 [file DataSheet1.docx]

# **Questionnaires for the LoMAS cohort study**

# Participant overview

Participant ID:

Center:

Midwife:

Registration date:

Recruitment date:

## Recruitment

### 1. Registeration

* Participant ID： * Participant’s name： *MW Contact Date： *Study ID：

### 2. Agreement

*I agree to take part in this study. Yes NO

1. I have been reading the informed consent, the researchers have explained the purpose, contents, risks and benefits of this research to me clearly. My questions so far have been answered. I understand the information printed on this form, and I volunteered for this study.

Yes NO

1. I agree to donate my biological samples (including blood, urine, hair, nails, buccal smear) and my baby's biological samples and my husband's oral samples for this research. I know our biological samples such as blood, urine and hair could be sent to study abroad, and I know the donation is voluntary. I could withdraw from the study whenever I decide, which won’t affect the normal antenatal care in the Chengdu Women's and Children's Central Hospital.

Yes NO

3. I agree the researchers to check on my medical records and personal information related to medicine. I know that my personal information will be kept secret. Yes NO

4. I agreed to the use of biological samples and personal information I donated in current and future scientific research (including commercial research and scientific research not related to this project). I know clearly that if this research could lead to new treatments and inventions of medical testing, I would not be able to gain commercial benefits from it.

Yes NO

5. I have the opportunity to invite my family or friends to help me to ask questions about this study, I know the person I should contact with if there is a problem. Yes NO

### 3. Enrolment

Group： ①Missed abortion ②Non-missed abortion

Inclusion Criteria：* D&C abortion

***** Able to provide written, informed consent

Exclusion Criteria: ***** with genetic diseases

***** with chronic metabolic diseases

***** ages < 16 or > 45

***** with twin pregnancy

### 4. Pregnant woman basic information

*name：

*date of birth：

*native place：

*Telephone number：

*address：

* Patient ID：

*Prenatal examination file number：

*education：1- primary school 2- high school 3- University 4- university or above 5- illiterate 6- other:

*occupation：

*blood type：

*height： cm

*weight before pregancy： kg

*smoke（1-no 2- smoking before 3-still smoking, /d 4-History of passive smoking, years） *alcohol（1-no 2- drinking before 3-still drinking, g/week）

*husband’s name：

*age：

*height： cm

*blood type：

*education：1- primary school 2- high school 3- University 4- university or above 5- illiterate 6- other:

*occupation：

*telephone number：

### 5. Obstetric History

*pregnancy（times）：_______（Including pregnancy, termination of pregnancy, abortion）

*delivery（times）：_______

* fetal malformations：①yes（add time）②no

* premature delivery：①yes（add time） ②no

*Stillbirth：①yes（add time） ②no

*tractus genitalis operation history（Cesarean section、obstetric forceps）：①yes（add time） ②no *spontaneous abortion：①yes（add time）②no

*artificial abortion：①yes（add time） ②no

*odinopoeia：①yes（add time） ②no

*acephalocystis racemosa：①yes（add time） ②no

* Gestational diabetes mellitus：①yes（add time） ②no

* intrahepatic cholestasis of pregnancy：①yes（add time） ②no

* Pre-eclampsia：①yes（add time） ②no

### 6. Current Pregnancy

* Last menstrual period：

* Gestational weeks：

*pregnancy mode：① Natural pregnancy ②IVF-ET ③ovulation stimulants

### 7. History of Present Illness

*colporrhagia：①yes ②no

*Viral infection：①yes ②no

*Exposure to radiation：①yes ②no

*hypermesis：①yes ②no

*fever：①yes ②no

* Long term exposure to poison：①yes ②no

* take medicine：①yes (add drug name and time) ②no

* anemia：①yes(add type) ②no

* take acyeterion before pregnancy 6 months：①yes（add drug name） ②no

other：

### 8. Past medical history

*heart disease：①yes ②no

*high blood pressure：①yes ②no

*nephritis：①yes ②no

*hepatitis：①yes ②no

*Tuberculosis：①yes ②no

* diabetes mellitus：①yes ②no

*blood disease：①yes ②no

* Mental illness：①yes (add type) ②no

*epilepsy：①yes ②no

*thyroid dysfunction：①yes ②no

*allergic history**：**①yes ②no

* operation history：①yes（add operation name and time） ②no

other：

### 9. Family History

*Twin history：①yes（add anyone）②no

* Gestational diabetes mellitus：①yes（add anyone）②no

* intrahepatic cholestasis of pregnancy：①yes（add anyone）②no

* Pre eclampsia：①yes（add anyone）②no

*neuropathy：①yes（add anyone）②no

*dementia：①yes（add anyone）②no

*malformation：①yes（add anyone）②no

*genetic disease：①yes（add anyone）②no

*high blood pressure：①yes（mother or father） ②no

* diabetes mellitus：①yes（mother or father） ②no

*Cardiovascular diseases：①yes（mother or father） ②no

* Immune diseases：①yes（mother or father） ②no

* Obstetric History：①yes（mother） ②no

*tumor history：①yes（mother or father） ②no

other：
